# Tuberculous Meningitis With Paradoxical Reaction in an Immunocompetent Young Male Treated by Interleukin-1 Receptor Antagonist

**DOI:** 10.1155/crdi/8891508

**Published:** 2025-11-03

**Authors:** Azade Kanat, Ayşin Kılınç Toker, Zuhal Özer Şimşek, Ali Koç, Ilhami Celik

**Affiliations:** ^1^Department of Infectious Diseases and Clinical Microbiology, Kayseri City Training and Research Hospital, Kayseri, Turkey; ^2^Department of Chest Diseases, Kayseri City Training and Research Hospital, Kayseri, Turkey; ^3^Department of Radiology, Kayseri City Training and Research Hospital, Kayseri, Turkey

**Keywords:** Interleukin 1 receptor antagonist protein, meningeal tuberculosis, *Mycobacterium tuberculosis*, paradoxical reaction

## Abstract

A 22-year-old man presented with headache, night sweats, intermittent fever, tremors, sleep disturbances, agitation, and hallucinations for 2 months. Thoracic computed tomography (CT) showed widespread interstitial nodular lesions, but initial cranial CT showed no significant pathology. Cerebrospinal fluid (CSF) analysis revealed 25 cells/mm^3^ of white blood cells (72% neutrophils), 83 mg/dL protein, and 30 mg/dL glucose (concurrent serum glucose was 103 mg/dL). Gram and Ziehl–Neelsen stains were negative for acid-fast bacilli. Tuberculosis (TB) cultures and Mycobacterium PCR tests remained negative. Biopsy of the bronchoalveolar lavage sample showed necrotizing granulomatous inflammation, and the *Mycobacterium tuberculosis* PCR result was positive. During the first month of first-line anti-TB treatment, the patient experienced recurrent severe headaches, persistent fever, and decreased visual acuity. Contrast-enhanced MRI revealed lesions that were consistent with tuberculous meningitis (TBM). Considering the possibility of drug-resistant TB, streptomycin 1 gr/qd (quaque die) intramuscularly and linezolid 600 mg/bid (bid in die) intravenously were added to the regimen. The patient's symptoms persisted during the second month of treatment. The patient experienced epileptic seizures. The control MRI showed an enlargement of the lesions. A paradoxical reaction was considered. Intravenous methylprednisolone 500 mg/day was initiated. The patient did not respond clinically, and his complaints continued. The patient was started on the IL-1 inhibitor anakinra. Paradoxical inflammatory reactions are common in TBM but challenging to predict. When severe, they can lead to significant neurological morbidity and death. This article aimed to share a case that did not respond to corticosteroids, a standard treatment for paradoxical reactions, but was successfully managed with the IL-1 inhibitor anakinra.

## 1. Introduction

Tuberculosis (TB), a global health threat with high mortality rates, is one of the oldest and most significant infectious diseases in human history. It is caused by bacteria from the *Mycobacterium tuberculosis* complex, which presents primarily in two clinical forms: pulmonary TB, which affects the lungs, and extrapulmonary TB, which can affect organs such as the pleura, lymph nodes, bones, joints, meninges, and gastrointestinal system [1].

A paradoxical reaction (PR) in TB treatment is defined as the clinical or radiological worsening of existing lesions or the appearance of new lesions in a patient despite effective anti-TB therapy and after ruling out other causes, such as drug resistance or treatment failure. PR is particularly common in cases of pulmonary, lymph node, and central nervous system (CNS) TB [2].

This report discusses a case of a young, immunocompetent, HIV-negative male with a severe and life-threatening form of tuberculous meningitis (TBM) who developed a PR during treatment. The severity of his condition, which required urgent and intensive management, underscores the critical nature of TBM and the need for prompt and effective treatment.

## 2. Case Presentation

A 22-year-old man presented with a 2-month history of worsening headaches and night sweats. He also reported intermittent fever, chills, and, more recently, sleep disturbances, agitation, and hallucinations. His personal and family histories were unremarkable; however, his occupation in livestock farming, a profession known for a higher risk of TB due to close animal contact, is crucial in understanding the potential sources of infection.

During the physical examination, the patient was conscious but agitated. According to relatives, his speech and movements had slowed. There were no signs of meningeal irritation or focal neurological findings.

The vital signs were as follows: temperature, 36.7°C; blood pressure,105/55 mmHg and heart rate, 72 bpm. Laboratory tests showed normal liver and kidney function, C-reactive protein of 0.8 mg/L (normal: 0–5 mg/L), white blood cell count of 8200/μL, neutrophil count of 6030/μL, hemoglobin 11.2 g/dL, and platelets 262,000/μL. Toxicology screening and HIV tests were negative.

**Figure 1:**
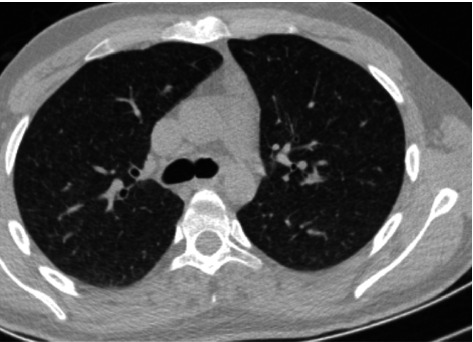
Thorax computed tomography (CT).

Thoracic computed tomography (CT) revealed widespread interstitial nodular lesions, while initial cranial CT was unremarkable (Figure 1). Cerebrospinal fluid (CSF) analysis showed white blood cells at 25 cells/mm^3^ (72% neutrophils), a protein level of 83 mg/dL, and a glucose level of 30 mg/dL (simultaneously, serum glucose was 103 mg/dL). Gram and Ziehl–Neelsen staining were negative for acid-fast bacilli. TB cultures and Mycobacterium PCR tests remained negative. Cytology from bronchoalveolar lavage showed necrotizing granulomatous inflammation.

After the initiation of the first-line anti-TB therapy (rifampicin, isoniazid, pyrazinamide, and ethambutol) along with dexamethasone at 0.15 mg/kg/qid (quater in die), the patient's condition improved significantly. On the third day of treatment, the patient was stable and transferred from the intensive care unit (ICU) to the ward, indicating a positive initial response to the therapy and instilling hope for his recovery.

**Figure 2:**
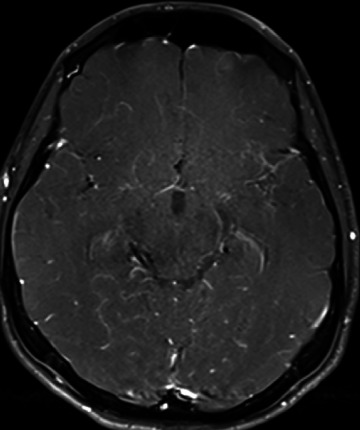
Axial contrast-enhanced T1W MR brain image taken at the initial admission shows contrast enhancement at the left sylvian fissure.

One month into treatment, the patient developed recurrent severe headaches, persistent fever, and decreased visual acuity. A contrast-enhanced MRI showed TBM lesions (Figure 2). Given the possibility of drug-resistant TB, streptomycin and linezolid were added to the regimen.

**Figure 3:**
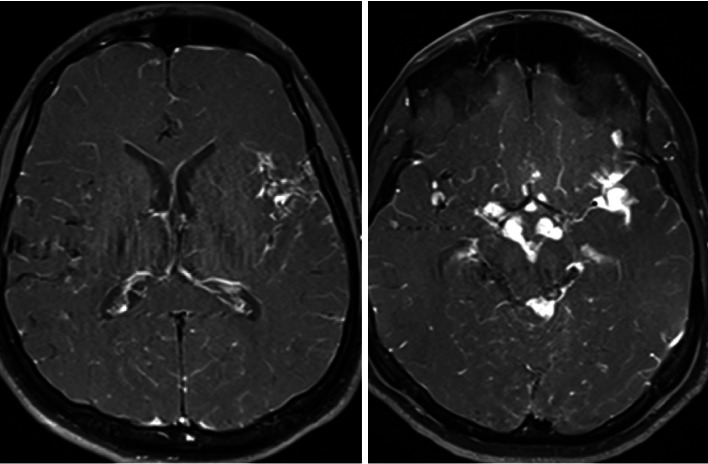
Axial contrast-enhanced T1W MR brain images show progressive contrast enhancement at the left sylvian fissure and basal cistern.

By the second month of treatment, the patient's symptoms persisted despite treatment. The patient developed epileptic seizures. Lesion growth was observed on repeat MRI (Figure 3). A PR was considered. Intravenous methylprednisolone 500 mg/day was started. The patient achieved partial clinical response, but his symptoms persisted. The IL-1 inhibitor anakinra was started in the patient.

**Figure 4:**
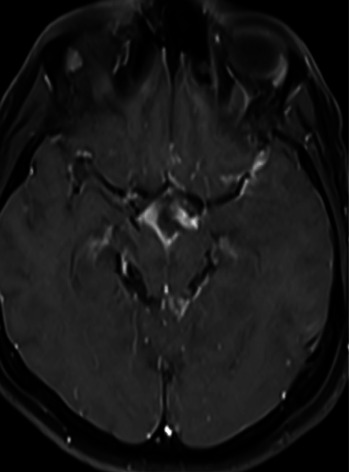
Axial contrast-enhanced T1W brain image taken after 1 month of medical therapy shows less prominent contrast enhancement at the sylvian fissure.

**Figure 5:**
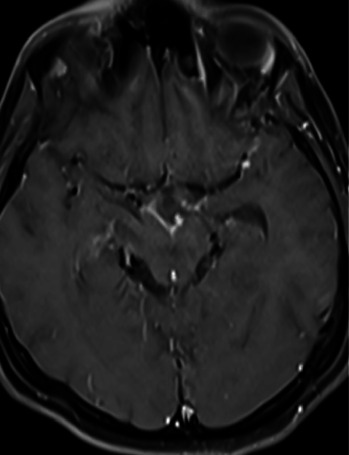
Axial contrast-enhanced T1W brain image after four months of medical therapy shows prominent regression of contrast enhancement at the left sylvian fissure.

The meticulous care and decision-making of the medical team were crucial for this successful outcome. Anakinra treatment was continued for 1 month. The patient was discharged with dual anti-TB therapy (rifampicin and isoniazid) and oral methylprednisolone (16 mg/day). Subsequent follow-ups did not observe additional pathology, emphasizing the accuracy of the medical team's decisions regarding the patient's recovery (Figures 4 and 5).

## 3. Discussion

TBM is a severe form of extrapulmonary TB that leads to significant morbidity and mortality, mainly due to its rapid course and neurological complications [3]. PRs during TB treatment, first described in the 1980s, are characterized by the worsening of existing lesions or the appearance of new lesions despite clinical improvement with anti-TB therapy [4]. In this case, our patient developed a PR after the first month of treatment. This is consistent with the findings from Singh et al., who reported that PRs typically occur within the first 3 months of therapy and are seen in 31% of TBM cases [5].

Radiologically, our patient exhibited increased lesion size on MRI, a common finding in TBM patients with PRs [6]. Studies by Rock et al. [7] have described similar imaging findings, including the enlargement of pre-existing lesions, the appearance of new tuberculomas, and hydrocephalus in CNS TB cases.

PRs are not uncommon in cases of TB meningitis. Tuberculomas are observed in two-thirds of cases evaluated by MRI and are symptomatic in half of patients [8]. Hydrocephalus, fever, headache, and visual disturbances are frequently accompanying symptoms. In our case, decreased visual acuity, severe headache, and fever were the most common findings associated with the PR.

The pathophysiology of PR is not fully understood, but it is thought to involve an exaggerated immune response to mycobacterial antigens following the initiation of effective anti-TB therapy [9]. Carvalho et al. proposed that this immune response is driven by proinflammatory cytokines, particularly TNF-α, which can exacerbate inflammation in the CNS [10]. Our patient's clinical course, with worsening symptoms after starting therapy, supports this hypothesis.

Corticosteroids are considered the mainstay of treatment for PR in TBM. They help reduce inflammation and stabilize the blood–brain barrier [3]. In this case, the patient was initially treated with methylprednisolone, but when this failed to fully control the reaction, anakinra, an IL-1 receptor antagonist, was introduced. Recent studies, such as those by van Arkel et al., have demonstrated the efficacy of anakinra in treating refractory PRs in TBM patients [11]. This aligns with our patient's significant clinical and radiological improvement after anakinra administration.

This case underscores the crucial importance and the weight of responsibility it places on medical professionals to consider PRs in all TBM patients, regardless of their immune status or the presence of HIV infection. This consideration is vital for effective management and improved patient outcomes.

## Data Availability

Data are available on request from the authors.
